# A Text-Book on Practical Obstetrics

**Published:** 1899-06

**Authors:** 


A TEXT-BOOK ON PRACTICAL OBSTETRICS. By EGBERT H. GRANDIN, M. D., Gynecologist to the Columbus Hospital; Consulting Gynecologist to the French Hospital; Late Consulting Obstetrician and Obstetric Surgeon of the New York Maternity Hospital, etc. With the collaboration of GEORGE W. JARMAN, M. D., Gynecologist to the Cancer Hospital; Instructor in Gynecology in the Medical Department of the Columbia University. Second edition; revised and enlarged. Illustrated; pp. xiv.—461. Philadelphia: The F. A. Davis Co. 1899.

This book in its second edition presents so much that is new that a somewhat detailed review is called for. It differs from older and larger treatises on obstetrics in that it assumes the reader to possess a sufficient knowledge of anatomy, physiology, pathology and embryology, and no attempt is made, therefore, to include these subjects in the work. Facts relating to them are only given when a proper understanding of the text demands them.

The volume is divided into four parts. Part I. deals with pregnancy, taking up the question of diagnosis, differential diagnosis, duration and hygiene of pregnancy, the pathology of pregnancy, and the diagnosis of presentation and position. In this section, as well as throughout the book, one notices the excellence of the illustrations. Those which are borrowed from older works on obstetrics are only such as can not be improved upon as elucidators of the text. The sixty-four full-page photographic plates, original with the authors, increase many fold the value of the book. Part II. discusses the subject of labor. The difficult topic of the mechanism of labor is presented with unusual clearness. Indeed, we rarely, if ever, have seen it better handled. The clinical course of labor, the management of normal and abnormal labor, and the care of the new-born infant, complete this section. The authors are opposed to the routine use of the Credé method of instilling silver-nitrate into the eyes of the new-born baby, and a large and respectful minority of obstetricians will doubtless agree with them.

In Part III. the normal and pathological puerperium are considered and Part IV. takes up obstetric surgery. In the latter chapter a section is devoted to obstetric asepsis and antisepsis which it hardly need be said is thorough and up-to-date. The comparatively new operation of symphyseotomy is handled as progressive, and at the same time conservative, surgeons might be expected to regard it; whatever it promises for the future its chief field for the present is the maternity hospital. Many obstetricians will doubtless differ from the authors as to the advisability of the immediate repair of the cervix as a routine procedure.

The faults of the book are few and of minor importance. Indeed, one of the most notable, that of positiveness in stating opinions, is hardly to be regarded as a failing. Taken as whole, it is one of the best works on obstetrics to be had. Clear, concise, without omitting anything essential, modern, well-illustrated, and well-printed, the volume will be welcome, not only to the young practitioner, who desires counsel where his limited experience fails him, but to the older man who, far from the centers of medical knowledge, desires to keep abreast with the times, but is too busy to have time to give to the perusal of elaborate treatises, burdened with their weight of anatomy, physiology and pathology. The work ranks in the field of obstetrics with Jacobi's in pediatrics, and this is high praise.

